# A novel ARHGAP family gene signature for survival prediction in glioma patients

**DOI:** 10.1111/jcmm.18555

**Published:** 2024-07-29

**Authors:** Jin Huang, Gaosong Wang, Jiahao Zhang, Yuankun Liu, Yifan Shen, Gengjing Chen, Wei Ji, Junfei Shao

**Affiliations:** ^1^ The Affiliated Wuxi People's Hospital of Nanjing Medical University, Wuxi People's Hospital, Wuxi Medical Center, Nanjing Medical University Wuxi Jiangsu China

**Keywords:** ARHGAP, glioma, machine learning, prognosis, tumour microenvironment

## Abstract

ARHGAP family genes are often used as glioma oncogenic factors, and their mechanism of action remains unexplained. Our research entailed a thorough examination of the immune microenvironment and enrichment pathways across various glioma subtypes. A distinctive 6‐gene signature was developed employing the CGGA cohort, leading to insights into the disparities in clinical characteristics, mutation patterns, and immune cell infiltration among distinct risk categories. Additionally, a unique nomogram was established, grounded on ARHGAPs, with DCA curves illustrating the model's prospective clinical utility in guiding therapeutic strategies. Emphasizing the role of ARHGAP30, integral to our model, its impact on glioma severity and the credibility of our risk assessment model were substantiated through RT‐qPCR, Western blot analysis, and cellular functional assays. We identified 6 ARHGAP family genes associated with glioma prognosis. Analysis using the Kaplan–Meier method indicated a correlation between elevated risk levels and adverse outcomes in glioma patients. The risk score, linked with tumour staging and IDH mutation status, emerged as an independent factor predicting prognosis. Patients in the high‐risk category exhibited increased immune cell infiltration, enhanced tumour mutational burden, more pronounced expression of immune checkpoint genes, and a better response to ICB therapy. A nomogram, integrating the risk score with the pathological features of glioma patients, was developed. DCA analysis and cellular studies confirmed the model's potential to improve clinical treatment outcomes for patients. A novel ARHGAP family gene signature reveals the prognosis of glioma.

## 1 INTRODUCTION

Derived from glial cells, gliomas are the predominant form of primary malignant tumours in the central nervous system, with annual incidence rates observed to be in the range of 3–6.4 per 100,000.^1^ According to the World Health Organization's classification, gliomas are divided into 4 grades (PMID: 35729337).WHO G2 and G3 are recognized as diffuse LGG, while WHO G4 is characterized as Glioblastoma (GBM).^2^ GBM, known for its aggressive nature, typically has a median survival duration of approximately 16 months, contrasting sharply with LGG, where patients may experience survival periods ranging from 1 to 15 years.^3^ GBM, being the predominant intracranial malignant primary tumour, constitutes 57% of all gliomas and 48% of primary malignant brain tumours.^4^ Despite advancements in conventional treatments like surgery, radiotherapy, and chemotherapy, the prognosis for these tumours remains bleak, primarily due to their highly infiltrative nature.^5^ Several molecular markers, such as mutations in isocitrate dehydrogenase (IDH)^6^ and co‐deletion of chromosomes 1 and 19 (1p/19q),^7^ have been utilized for molecular pathological diagnosis, treatment selection, and prognosis evaluation. Nonetheless, these markers have limited impact on the effective management of gliomas in clinical settings. Current treatment options for GBM, such as surgery, radiation therapy, chemotherapy, and targeted therapy, have limitations including high surgical risks, severe side effects of radiation and chemotherapy, and limited specificity and the efficacy of targeted therapy. Therefore, identifying new biomarkers and developing innovative treatment strategies are crucial to overcome these limitations, improve treatment outcomes, reduce side effects, and enhance the quality of life for patients with GBM. Therefore, identifying novel biomarkers for better prognostic prediction of glioma is an imperative need. Meanwhile, molecular targeted therapy and immunotherapy techniques are rapidly developing in other malignancies, which are the future direction of glioma.

Tumour metastasis is the process of migration of malignant tumour cells from their primary focus to other parts of the body. Cellular migration, a key factor in tumour progression, significantly contributes to the mortality of tumour patients.^8^, ^9^ This process is crucial for both the metastasis of tumours and the immune response. Maintaining cell polarity is essential for directed movement.^10^ The dynamic restructuring of the actin cytoskeleton plays a pivotal role in cell polarization. Assisted by actin, RHO GTPases that bind GTP can interact with various effector proteins, such as PAK, Was, and Nck1, to modulate cellular activities including the secretion, phagocytosis of apoptotic cells, and polarization of epithelial cells.^11^, ^12^

Rho GTPases play a pivotal role in several critical cellular processes, including the remodelling of the actin cytoskeleton, adherence of cells, mobility of cells, transportation of vesicles, activation of transcription, regulation of gene expression, and control of the cell cycle.^13^ When the level of Rho GTPase protein is altered, abnormal Rho signalling occurs, which affects cytoskeleton reorganization and cell migration.^12^ RHO GTPases act as molecular switches that control multiple cellular processes by influencing the switching of the GDP/GTP binding state.^12^ The negative regulator of RHO GTPase, GTPase‐activating protein (RhoGAP), is also known as the ARHGAP family.^14^ The ARHGAP family plays diverse roles in various types of tumours. ARHGAP43 is overexpressed in patients with acute myeloid leukaemia, and the downregulation of ARHGAP43 expression inhibits the proliferation of AML cells.^15^ Additionally, the ARHGAP‐RhoA signalling pathway causes homotypic adhesion‐triggered cell death in metastatic diffuse‐type gastric cancer.^16^ However, the role of ARHGAPs as a pro‐carcinogenic factor in glioma has not been studied, especially in the immune microenvironment, and the mechanism of action has not been elucidated.

This research centered on evaluating the prognostic significance of the ARHGAP gene family in glioma. We established an ARHGAP‐centric prognostic score model to delve into the variations within the tumour microenvironment among patients categorized by this risk scoring system. Employing a 6‐gene signature we had formulated, the prognostic and immunological attributes across different risk categories were assessed. Notably, in our model, the elevated expression of the ARHGAP30 gene in gliomas was linked to increased cellular invasiveness, as evidenced by cellular functional assays. This study aims to investigate the correlation between the survival of glioma patients and six genetic features, with the objective of enhancing the precision of personalized treatment strategies for clinical practice. We anticipate that by analysing these genetic features, we will be able to improve the accuracy of prognosis prediction and develop more effective treatment plans for patients at high risk.

## 2 MATERIALS AND METHODS

### 2.1 Gene expression and clinical data acquisition

In this study, extensive RNA sequencing and mutation data, along with detailed clinicopathological features for the TCGA‐LGGGBM cohort, were meticulously retrieved from the University of California, Santa Cruz's Xena platform. The TCGA repository contained a comprehensive collection of 703 glioma samples. For the purpose of validating our model, gene expression profiles of 693 glioma patients were systematically extracted from the China Glioma Genome Atlas (CGGA) portal. In order to establish a robust baseline for comparison, control samples from healthy individuals were sourced from the GTEx project. All the gene expression data utilized in this study were presented in the TPM format. We used the ‘limma’ and ‘sva’^17^ software packages in order to make sure that the integration of diverse datasets was done correctly and dependably. These software packages are well known for their efficiency in correcting and harmonizing data in batches. Before analysis, the data has been appropriately standardized and pre‐processed, including the handling of missing values and the detection and management of outliers. The standardization process employs Z‐score normalization to ensure comparability between different datasets.

### 2.2 Functional enrichment analysis

The first step is to download the dataset and then perform functional enrichment analysis. To do this, we browsed through various databases where we got to know of MSigDB and downloaded their database. Now we are ready to perform Gene Set Enrichment Analysis (GSEA). We used GSVA from the R package to conduct meticulous enrichment analysis and found accordingly.^18^ Moreover, to enhance the analysis of the gene functions, the authors performed extensive gene enrichment assessment which included evaluating both KEGG and GO categories. They utilized the cluster Profiler package in R for this purpose and the results showed a comprehensive and extensive exploration of the gene functions.

### 2.3 Consensus clustering

We used the consensus clustering method to find out how many groups were present in our dataset. We selected the consensus clustering method for this investigation. By applying the ‘ConsensusClusterPlus’ package^19^ in R through the use of genetic markers, the different groups of glioma patients were successfully separated from the TCGA and CGGA datasets. Utilizing consensus clustering, a matrix with *k* = 2 revealed distinct groupings. Using the k‐means method, the clustering process is repeated 1000 times to ensure the confidence of classification. Based on the consensus index and cumulative distribution function (CDF), the optimal number of clusters is determined. The heat map gave us a picture of how the results looked like. We used graphs and CDF plots to figure out how many clusters we should have.

### 2.4 Development and validation of prognostic signatures

In order to minimize the impact of the variation between two specific data sets, the author constructed our initial analysis model utilizing the svA package within R. The resultant model was executed via an LS regression procedure. We then filtered the Ta Southern cohort by selecting only those variables that displayed a *p* < 0.001 in a univariate Cox study. The ‘glmnet’ package in R was utilized to streamline the gene count in the ultimate risk model. Subsequently, genes identified through LASSO regression were analysed using multivariate Cox regression, structured by the formula: risk score = Ʃ (ð × Expi), where ð denotes the regression coefficient and Expi is the expression level of each mRNA. Patients were classified into high‐risk and low‐risk categories based on their median risk score. Survival analysis and risk stratification maps were created using the ‘survminer’ and ‘ggrisk’ packages in R, illustrating the survival variances and individual patient statuses. Additionally, the CGGA cohort served as an independent external validation set to ascertain the efficacy of our prognostic model.

### 2.5 Estimation of the tumour immune microenvironment of the prognostic signature

The CIBERSORT and ssGSEA R scripts were utilized to determine the relative proportions of infiltrating immune cells in the study.^20^ The CIBERSORT algorithm was used to analyse the key population of immune cells in the two groups with diverse life possibilities. In each sample, we assigned equal proportions of estimated immune cell types, where the sum of all proportions was equal to one. Furthermore, we completed a Spearman's rank correlation analysis to explore the links between the levels of risk scores and the existence of immune system cells.

### 2.6 Construction and evaluation of a predictive nomogram

The research team developed a detailed roadmap, which was able to incorporate same risk factors with the deadly diseases related details and give out with the prognosis late estimation process. The model's precision was confirmed through graphs that are very important in making sure the model works correctly Note that the calibration plots are essential for verifying the precision of the model and doing internal validation. The initiative we undertook was to implement DCA to discern the clinically beneficial outcomes and overall benefit of our model.^21^ And to hopefully understand how good our model will be on predictions, we were able to generate ROC curves using the ‘timeROC’ package in R. Furthermore, the packages gave us some measures regarding how well the model is likely doing and how well it'll fare in predicting 1, 3 and 5 years' survival rates for patients affected by LGG.

### 2.7 Tumour immune single cell hub database

The Tumour Immuno‐Single Cell Center The Tisch (TISCH) website serves as a comprehensive repository, providing gene expression analysis within the TME environment for single‐cell RNA sequencing data.^22^ This platform offers a thorough examination of the diversity present in the TME, through the use of various datasets and cell types.

### 2.8 Cell culture and transfection

Human glioma lines U87, T98G, U118, U251, and HA were cultured in DMEM with 10% FBS and penicillin/streptomycin, at 37°C and 5% CO2. ARHGAP30 was overexpressed using a GV492‐based vector (Genechem), with CON335 as control.

### 2.9 Western blot analysis

Initially, proteins were extracted from cells utilizing RIPA buffer (Cell Signalling Technology, 9806, USA). Protein concentrations were determined, followed by loading 20 μg of protein onto 8% SDS‐PAGE gels for separation. Proteins on PVDF membranes were blocked with 5% milk, incubated with primary antibodies, followed by PBST washes and secondary antibody treatment. Detection used WesternBright ECL (Advansta) and ChemiDoc™ XRS C (Bio‐Rad), with primary antibodies ARHGAP30 (Invitrogen) and GAPDH (Proteintech).

### 2.10 RT q‐PCR

Total mRNA was isolated utilizing TRIzol reagent (Sigma–Aldrich, T9424, USA). The synthesis of cDNAs was performed using the PrimeScript RT‐PCR kit (Takara, RR047, Japan). Quantitative real‐time PCR was executed with SYBR Premix Ex Taq™ II (Takara, RR047, Japan) in an ABI StepOnePlus Real‐Time PCR System. Primers for PCR were sourced from Sheng Kong Company in Shanghai, China. Gene expression levels were normalized against GAPDH. The PCR primers synthesized for ARHGAP30 were as follows: (5′–3′): Forward: GGAGGAGTATGGAGTGGTGGATGG. Reverse: TGGCTTCCGCTCTGACTCAAATTC. GAPDH primers (5′–3′): Forward: ATCTCTGCCCCCTCTGCTGA. Reverse: GATGACCTTGCCCACAGCCT.

### 2.11 Cell counting kit‐8 assay

Initially, 1000 cells per well were plated in a 96‐well plate and incubated at 37°C with 10 μL of CCK‐8 reagent (Dojindo, CK18, Japan) for 4 h. Cell enumeration was conducted using an ELx800 plate reader (Termo, MultiskanSpectrum, USA) by assessing the absorbance at 450 nm. The proliferation of cells was represented by the fold increase in cell count from Day 0 to Day 4, which was then graphically depicted.

### 2.12 Migration assay

The migratory activity of cells was assessed using a Boyden chamber equipped with a gelatin‐coated polycarbonate filter (pore size = 8 μm). Briefly, 10,000 cells were placed in the upper chamber, while the lower chamber was filled with 500 μL of 10% FBS medium. Following a 24‐h incubation period, cells that migrated to the other side of the membrane were stained with 0.1% crystal violet and then fixed with 4% paraformaldehyde (PFA). The invasive potential of the cells was determined by counting the total number of cells under a microscope.

### 2.13 EdU assay

To evaluate cell proliferation in both control and treated groups, the EdU assay was implemented. Cells were incubated with EdU (Invitrogen, C10639, Canada) for 1 h at 37°C at a concentration of 10 μM. Post‐incubation, the medium was discarded, and the remaining cells were fixed with 4% paraformaldehyde at room temperature for 30 min, followed by permeabilization with 1% Triton X‐100 (Biosharp, BS084, China) for 20 min. Subsequently, cells were stained as per the manufacturer's instructions and visualized using a fluorescence microscope (Leica Microsystems GmbH, Mannheim, Germany). Image composition and cell enumeration were conducted using Fiji ImageJ software. The growth rate and proliferation were deduced by dividing the count of EdU‐positive cells by the number of DAPI‐positive cells.

### 2.14 Statistical analysis

Statistical analyses were conducted using R (v4.1.1) and its extensions. Kaplan–Meier and log‐rank tests evaluated survival across patient groups. Wilcoxon and Kruskal–Wallis tests assessed continuous variables in two or more groups, respectively. Cox regression in R's ‘survival’ package identified prognostic factors, with *p* < 0.05 indicating significance. Significance levels indicated as *p < 0.05, **p < 0.01 and ***p < 0.001.

## 3 RESULTS

### 3.1 Identification of prognosis‐related ARHGAP family genes

**FIGURE 1:**
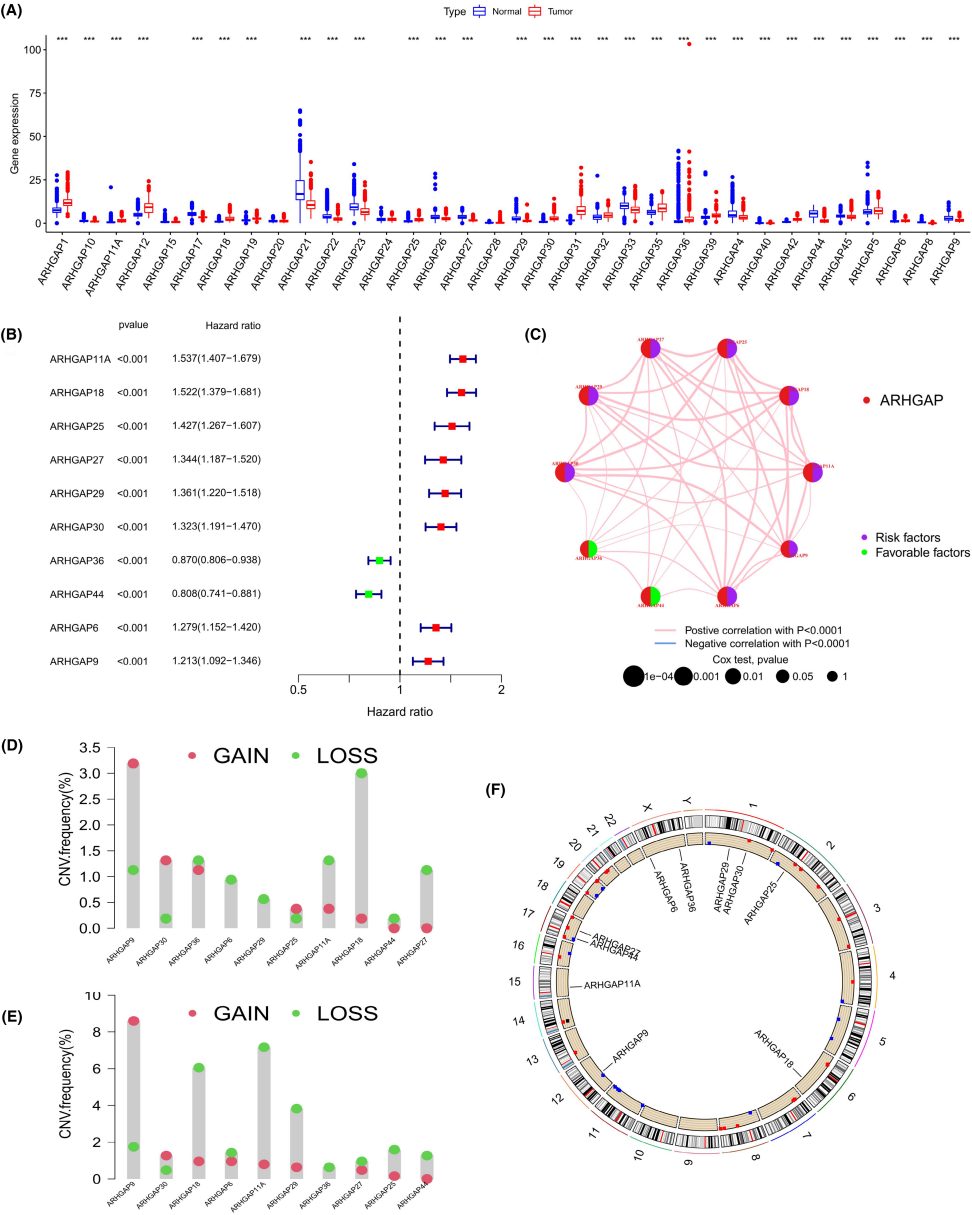
Analysis of ARHGAP family genes in glioma context. (A) Comparative analysis revealing expression variations of 35 ARHGAP family genes between glioma and normal tissue samples. (B) A forest plot depicting the prognostic impact of ARHGAP family genes, determined through univariate Cox regression analysis within the CGGA cohort, highlighting statistically significant genes (*p* < 0.001). (C) A network visualization illustrating the interconnections and relationships among the 10 ARHGAP family genes of interest. (D, E) Illustrations of copy number variations (CNVs) observed in these 10 ARHGAP family genes. (F) Diagrammatic representation mapping the chromosomal locations of these 10 ARHGAP family genes, highlighting their genomic distribution. ***p < 0.001.

In the TCGA and CGGA cohorts, a comprehensive analysis of 35 ARHGAP family genes was conducted. Remarkably, in the TCGA‐LGG and GTEx cohorts, a significant majority of the ARHGAP family genes, with the exception of ARHGAP15, ARHGAP20, ARHGAP24, and ARHGAP28, exhibited notable differential expression between tumour tissues and normal samples, as established through Wilcoxon test analysis (Figure 1A). Within the CGGA dataset, univariate Cox regression analysis highlighted that 10 ARHGAP family genes were significantly linked to the survival rates of glioma patients, with a statistical significance threshold set at *p* < 0.001 (Figure 1B). The network diagrams meticulously detailed the correlations between expression levels of these 10 ARHGAP genes and their potential impact on enhancing or diminishing patient survival prospects (Figure 1C). Copy number variations (CNVs) significantly impact the prognosis and treatment of gliomas. Certain CNVs, such as increased MET gene copies, can enhance tumour growth and metastasis, while CNVs in ARHGAP family genes can lead to abnormal ARHGAP expression, affecting patient outcomes and treatment response. This research direction opens up opportunities for the development of targeted therapies against ARHGAPs. Acknowledging the frequent chromosomal alterations in glioma patients,^23^ CNV data from the TCGA database were meticulously examined to delve deeper into the chromosomal changes affecting these ARHGAP genes in LGG (Figure 1D) and GBM patients (Figure 1E), pinpointing the specific chromosomal locations of each gene (Figure 1F). Notably, the most significant changes in ARHGAP9 were observed on chromosome 12, while ARHGAP18 and ARHGAP11A predominantly experienced losses on chromosomes 6 and 15 respectively.

### 3.2 Consistent clustering using 10 ARHGAP genes in glioma

**FIGURE 2:**
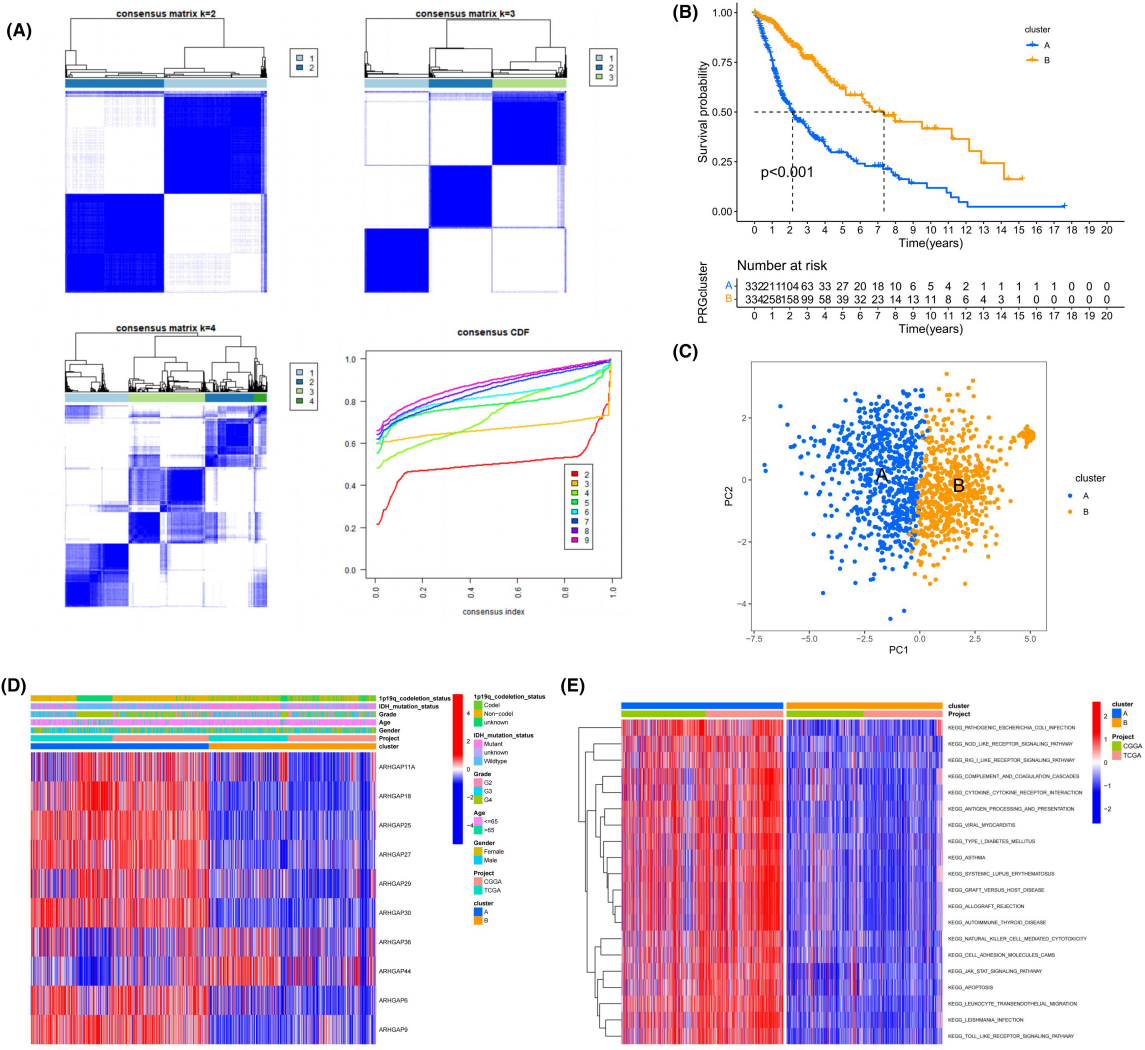
Categorization of glioma into subgroups based on ARHGAP family genes. (A) Utilizing consensus clustering, a matrix with *k* = 2 revealed distinct groupings. (B) Kaplan–Meier survival curves depicting significantly different survival outcomes between the identified subtypes (*p* < 0.001). (C) Principal component analysis (PCA) effectively segregating two subtypes, predicated on the expression profiles of 10 ARHGAP family genes. (D) A heatmap presentation of gene expression levels of the ARHGAP family and corresponding clinicopathological attributes for each subtype. (E) Gene Set Variation Analysis (GSVA) delineating divergent enrichment patterns in the KEGG pathways between clusters A and B.

To gain a deeper insight into the role of these 10 ARHGAP family genes in glioma, we conducted comprehensive consensus clustering based on univariate Cox analysis results using the Consensus Cluster Plus package in R. The clustering, as illustrated in Figure 2A, revealed that at *k* = 2, the CDF curve flattened, indicating an effective division of the cohort into two distinct subtypes. The survival analysis comparing these two subtypes demonstrated a marked difference in prognosis, as evidenced by a significant *p*‐value (*p* < 0.001, Figure 2B). Principal component analysis (PCA) was utilized to validate the robustness of this subtype classification, confirming distinct separation at *k* = 2 (Figure 2C). Heatmaps illustrating ARHGAP family gene expression and associated clinicopathological characteristics for each subtype underscored the potential of these genes as key prognostic markers in glioma (Figure 2D). Beyond assessing the distribution of the 33 ARHGAP family genes in these clusters, we employed the GSVA package to scrutinize the differential enrichment in KEGG pathways between clusters A and B, acknowledging their notable divergence (Figure 2E). Cluster A, characterized by a poorer prognosis, predominantly encompassed pathways related to immune disorders, such as autoimmune thyroid disease, systemic lupus erythematosus, and transplant rejection, potentially shedding light on the mechanisms of immune dysfunction and escape during tumorigenesis.

### 3.3 Immune infiltration and differential gene expression in the two subtype clusters

**FIGURE 3:**
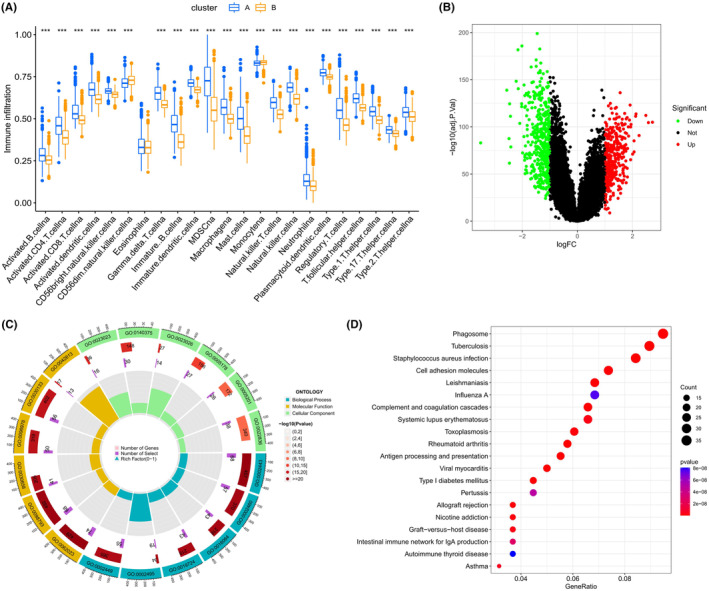
Variances in gene expression and immune infiltration across glioma subtypes. (A) Displays the immune cell infiltration profile for each glioma subtype, illustrating distinct immune landscapes. (B) A volcano plot illustrating genes with significant upregulation and downregulation in each subtype. (C) A circular representation of Gene Ontology (GO) terms and their enrichment analysis in differentially expressed genes (DEGs). (D) A bubble chart depicting the Kyoto Encyclopedia of Genes and Genomes (KEGG) pathway enrichment analysis outcomes for the identified DEGs. ***p < 0.001.

The differences in immune cell infiltration levels between the two subgroups were depicted using boxplots, employing the ssGSEA methodology. Intriguingly, it was observed that the infiltration percentages of almost all immune cells were higher in subgroup A compared to subgroup B, with the exception of eosinophils (Figure 3A). Volcano plots representing differential analyses of these subgroups are presented in Figure 3B. Enrichment analyses for GO and KEGG pathways were conducted on these differentially expressed genes (DEGs), revealing associations with various factors. These included ‘leukocyte‐mediated immunity’ within the biological process (BP) category, ‘extracellular matrix’ in the cellular component (CC) category, and ‘immune receptor activity’ in the molecular function (MF) category. KEGG pathway analysis indicated that these genes were linked to immune‐related diseases such as ‘*Staphylococcus aureus* infection’ and ‘tuberculosis,’ among others (Figure 3C,D).

### 3.4 Construction of an ARHGAP family genes‐related prognosis signature

**FIGURE 4:**
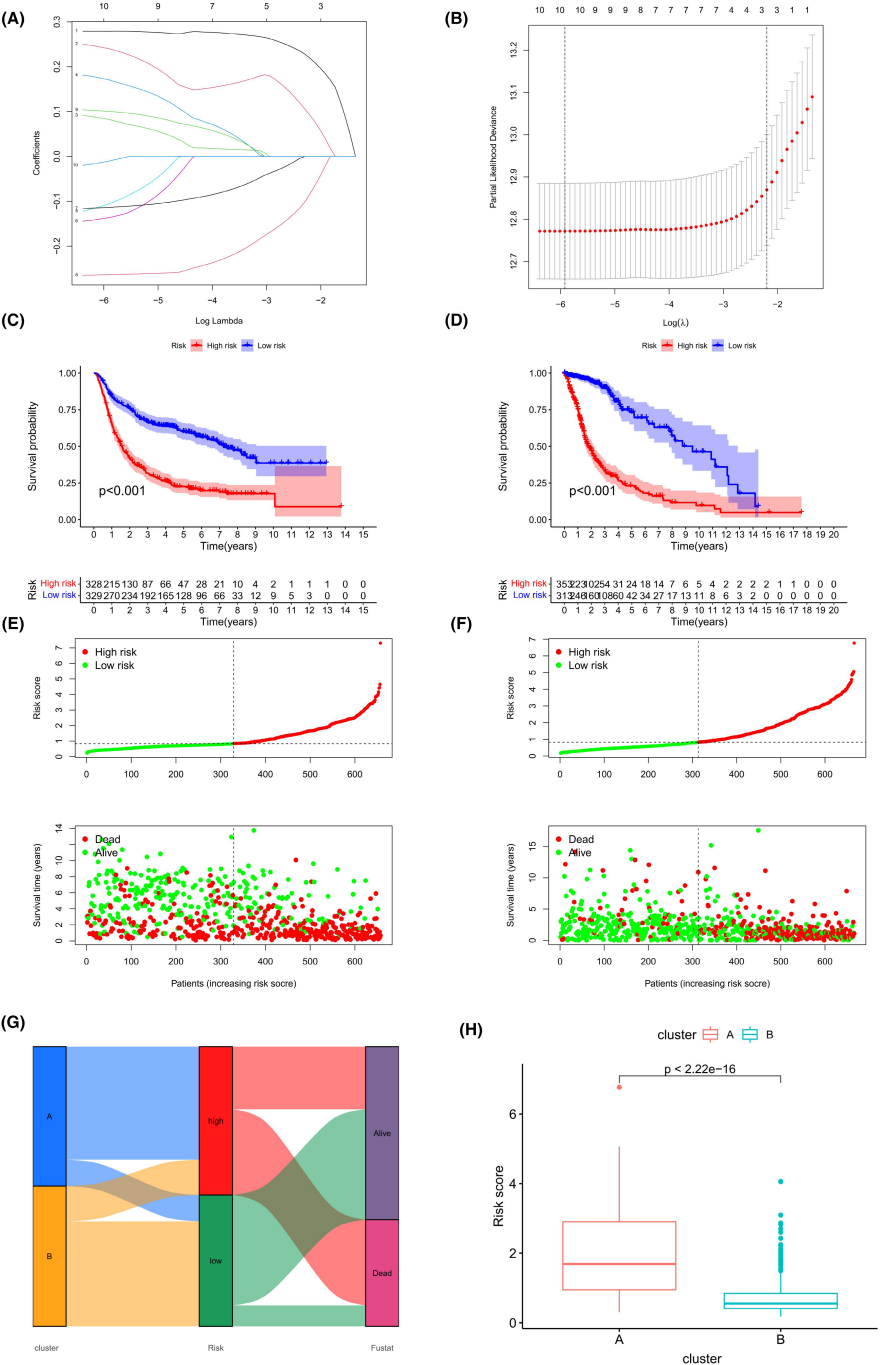
Prognostic analysis using ARHGAP family genes. (A) The LASSO model's parameter optimization via 10‐fold cross‐validation, with each gene represented by a distinct curve. (B) Co‐efficient profile analysis in the LASSO model, with vertical lines indicating the optimal lambda value. (C, D) Kaplan–Meier survival curves in CGGA and TCGA cohorts, comparing survival outcomes between high‐ and low‐risk groups. (E, F) Risk plots showing survival outcomes and risk score distribution for individual patients in the CGGA and TCGA cohorts. (G) Alluvial diagrams depicting transitions among risk groups, subgroups, and survival outcomes. (H) Risk score comparison for the two glioma subgroups established based on ARHGAP family gene analysis.

To evaluate the clinical significance of ARHGAP family genes, Lasso‐penalized Cox regression analysis was conducted on a set of 10 genes, with a significance level of *p* < 0.05 (Figure 4A,B). Subsequently, multivariate Cox regression analysis identified 6 ARHGAP genes—ARHGAP11A, ARHGAP18, ARHGAP27, ARHGAP30, ARHGAP36, and ARHGAP44—as independent prognostic indicators. We derived a risk score formula based on their coefficients: Risk score = 0.269*expression of ARHGAP11A* + 0.231expression of ARHGAP18 + 0.245*expression of ARHGAP27*–0.132expression of ARHGAP30–0.099*expression of ARHGAP36*–0.290expression of ARHGAP44. Kaplan–Meier curves revealed that the high‐risk group in the CGGA cohort had a significantly poorer prognosis, a finding corroborated in the TCGA validation cohort (Figure 4C,D). Risk stratification plots provided detailed survival analyses for individual patients within the CGGA and TCGA cohorts, with the high‐risk group predominantly exhibiting poor prognoses (Figure 4E,F). The alluvial plots demonstrated the interplay among clustering, risk levels, and survival outcomes in relation to ARHGAP gene family (Figure 4G), highlighting notably higher risk scores and poorer prognoses in subgroup A (Figure 4H).

### 3.5 Immune infiltration in different risk groups

**FIGURE 5:**
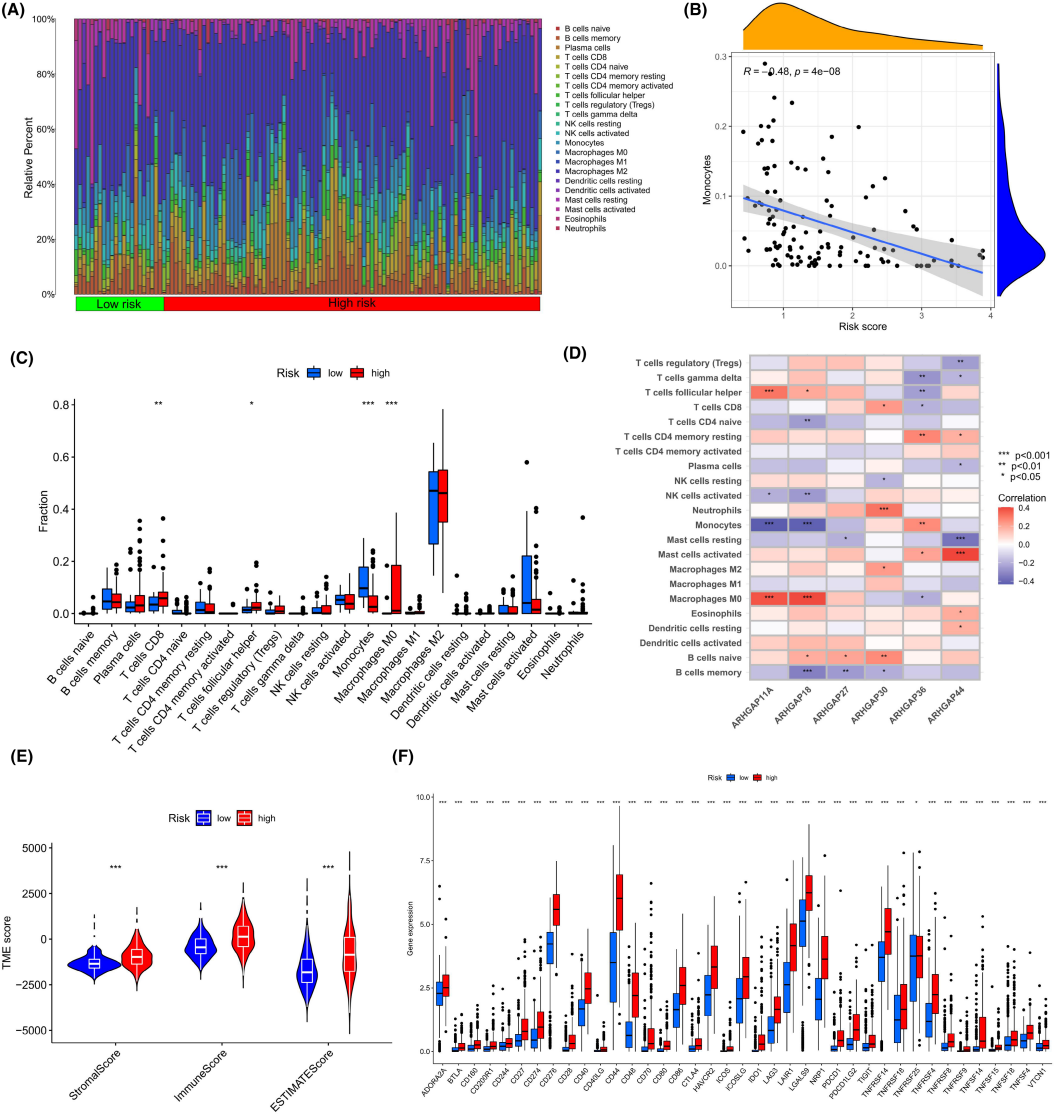
Immune landscape in glioma based on Risk Scores. (A) Comparative analysis of immune cell infiltration across varied risk subgroups. (B) Exploration of the relationship between risk scores and CD8^+^ T cell abundance in glioma samples. (C) Interplay of immune cells with 6 selected ARHGAP family genes. (D) Contrasting immune cell infiltration patterns in high‐risk versus low‐risk groups. (E) Expression profile analysis, comparing high‐risk and low‐risk groups. (F) Examination of immune checkpoint gene expression across risk categories.

The role of the immune microenvironment in glioma progression and the efficacy of immunotherapeutic approaches is critical. In this regard, an in‐depth analysis of the TME in glioma patients was conducted. The relative proportions of infiltrating immune cells in both high‐ and low‐risk groups were quantified using the CIBERSORT method. Initially, glioma samples were ranked according to risk scores, from lowest to highest, revealing the distribution of diverse immune cells (Figure 5A). A trend was observed where the percentage of activated monocytes decreased in correlation with increasing risk scores (*R* = −0.48, Figure 5B). Notably, the high‐risk group showed greater infiltration by CD8^+^ T cells and M0 macrophages (Figure 5C), suggesting that their enhanced activation might contribute significantly to poor prognoses in glioma. The 6 genes that were pivotal in calculating the risk score exhibited strong associations with the infiltration of various immune cells (Figure 5D), with ARHGAP30, in particular, showing significant correlation with CD8^+^ T cell infiltration. Additionally, an analysis of the expression profiles indicated higher stromal and immune scores in the high‐risk group (Figure 5E). Lastly, the expression levels of immune checkpoint genes across different risk score groups were evaluated in Figure 5F, revealing increased expressions in the high‐risk group, implying that patients in this group might be more responsive to immune checkpoint blockade (ICB) therapies.

### 3.6 Establishment of a prognostic nomogram for glioma patients

**FIGURE 6:**
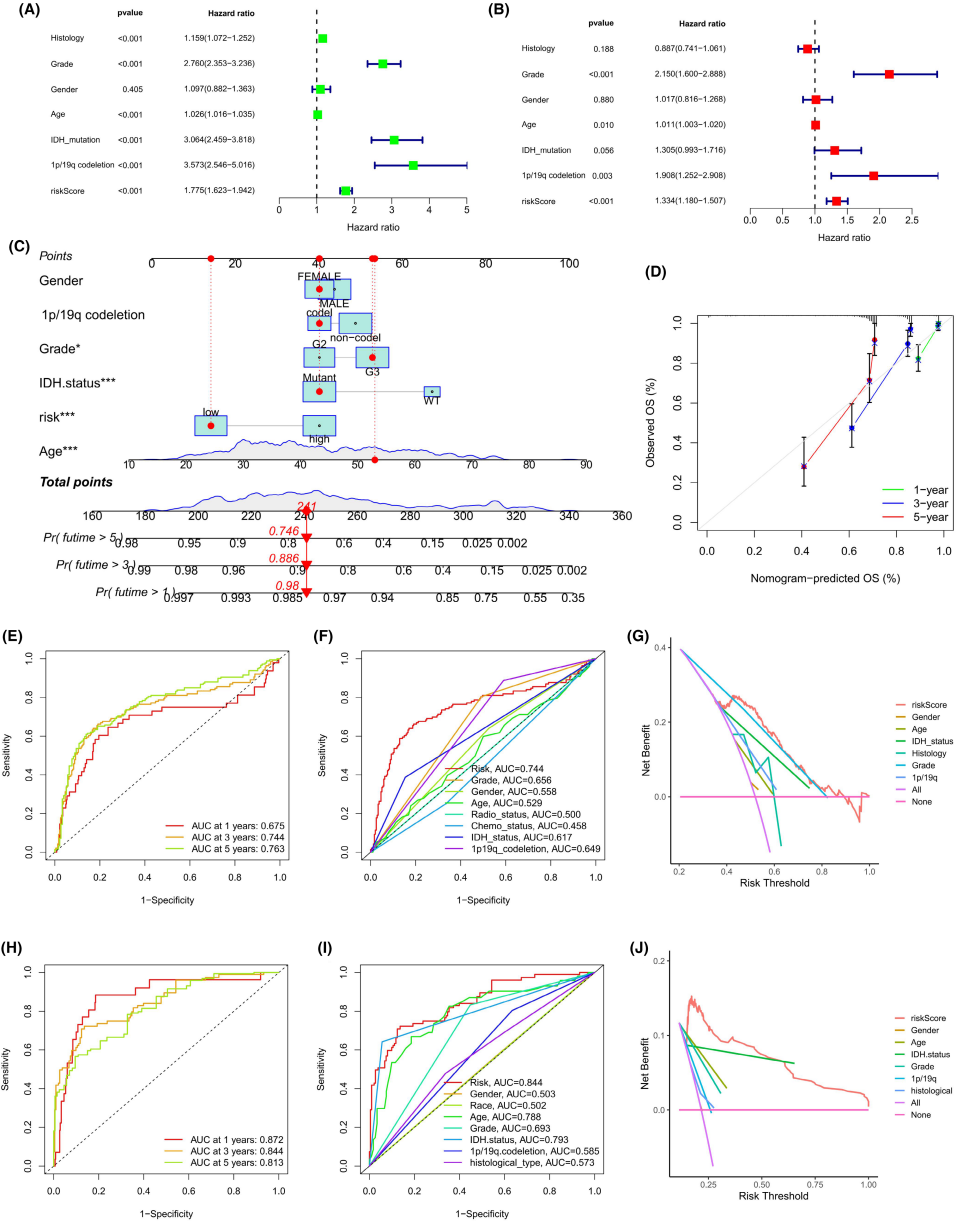
Evaluation of Risk Score's prognostic relevance in glioma. (A) Univariate, and (B) multivariate Cox regression analyses in the TCGA cohort, examining prognostic significance and clinical factors (age, race, gender, IDH mutation, 1p/19q co‐deletion). (C) A predictive nomogram integrating risk score and clinical parameters for 1‐, 3‐, and 5‐year survival predictions. (D) Calibration plots comparing predicted outcomes with actual survival data over 1, 3, and 5 years. (E) Time‐dependent AUC values for risk scores in TCGA and (H) CGGA cohorts across different time points. (F) Comparative AUC values for risk scores and clinical factors at 3 years in TCGA and (I) CGGA cohorts. (G) DCA curves assessing the clinical utility of risk scores and clinical factors at 3 years in TCGA and (J) CGGA cohorts.

**FIGURE 7:**
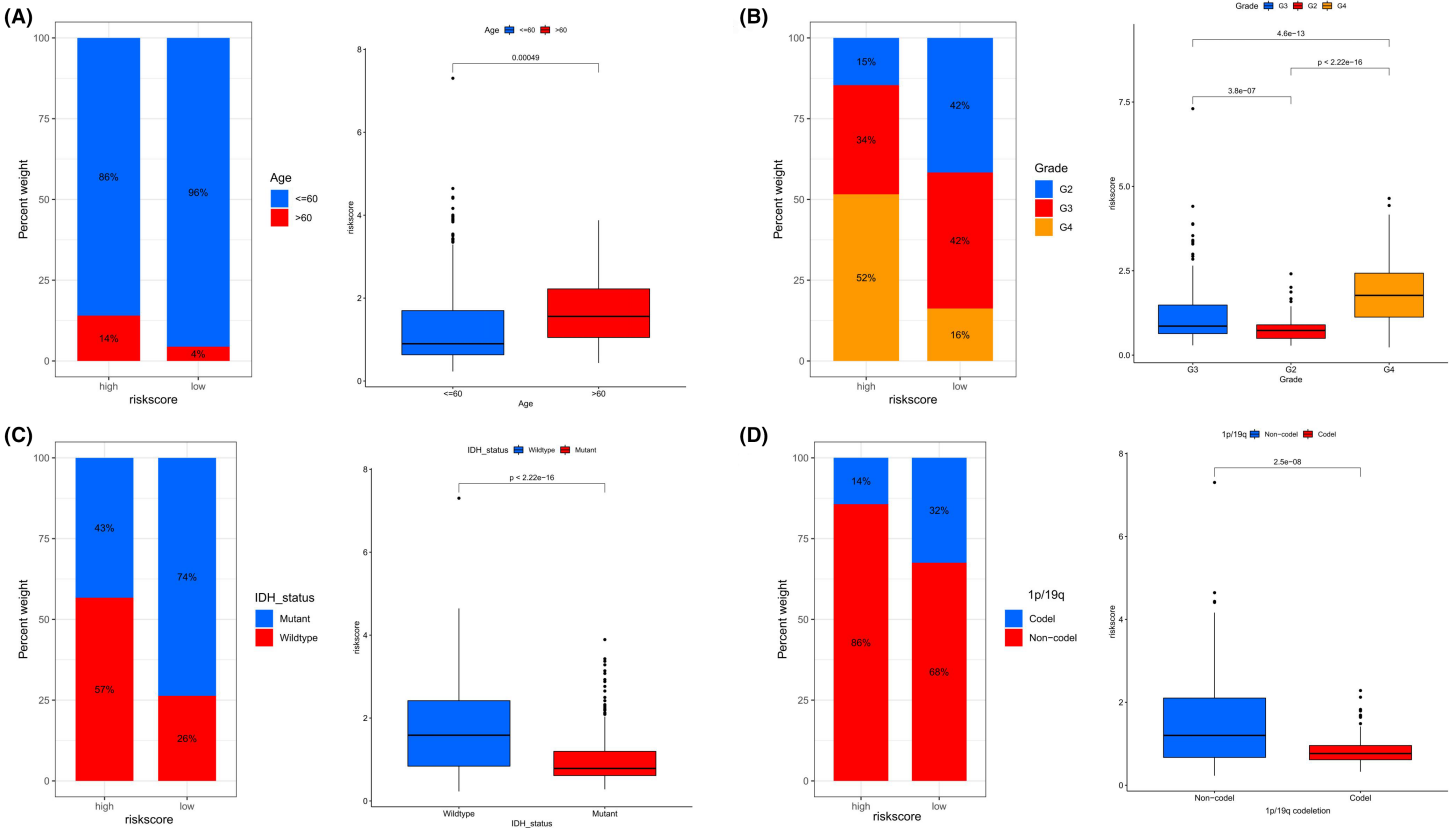
Variability in Risk Scores across clinical characteristics subsets in the TCGA Cohort. (A) Discrepancies in risk scores with respect to patient age groups. (B) Variations in risk scores corresponding to tumour grades. (C) Fluctuations in risk scores based on IDH mutation statuses. (D) Divergences in risk scores in relation to 1p/19q co‐deletion. Significance levels indicated as *p < 0.05, **p < 0.01, ***p < 0.001.

In the CGGA cohort, both univariate and multivariate Cox regression analyses established that the risk score was a key independent prognostic factor for glioma patients (Figure 6A,B). Subsequently, we integrated various prognostic influences such as ages, tumour grades, IDH mutation status, 1p/19q codeletion status, and risk classification into a nomogram (Figure 6C). Calibration plots were developed to verify the correlation between the predicted and actual overall survival (OS) based on the prognostic model, demonstrating the model's accuracy (Figure 6D). Additionally, within the CGGA cohort, the risk classification effectively predicted OS, with the AUC values for 1‐, 3‐ and 5‐year OS being 0.675, 0.744, and 0.763, respectively (Figure 6E). Comparable outcomes were observed in the TCGA cohort, with AUC values for 1‐, 3‐ and 5‐year OS being 0.872, 0.844, and 0.813, respectively (Figure 6H). Notably, the three‐year AUC for the risk subgroups in both the CGGA and TCGA cohorts surpassed that of other clinicopathological features (0.744 and 0.844; Figure 6F,I). The three‐year decision curve analysis (DCA) revealed that the risk score efficiently predicted survival in glioma patients (Figure 6G,J). Based on these findings, we meticulously compared risk scores across various clinical characteristic subgroups. It was observed that patients older in age, with stage G4, lacking IDH mutations, and without 1p/19q codeletions, tended to have higher risk scores (Figure 7A–D, all *p* < 0.05).

### 3.7 Immunotherapy and mutation

**FIGURE 8:**
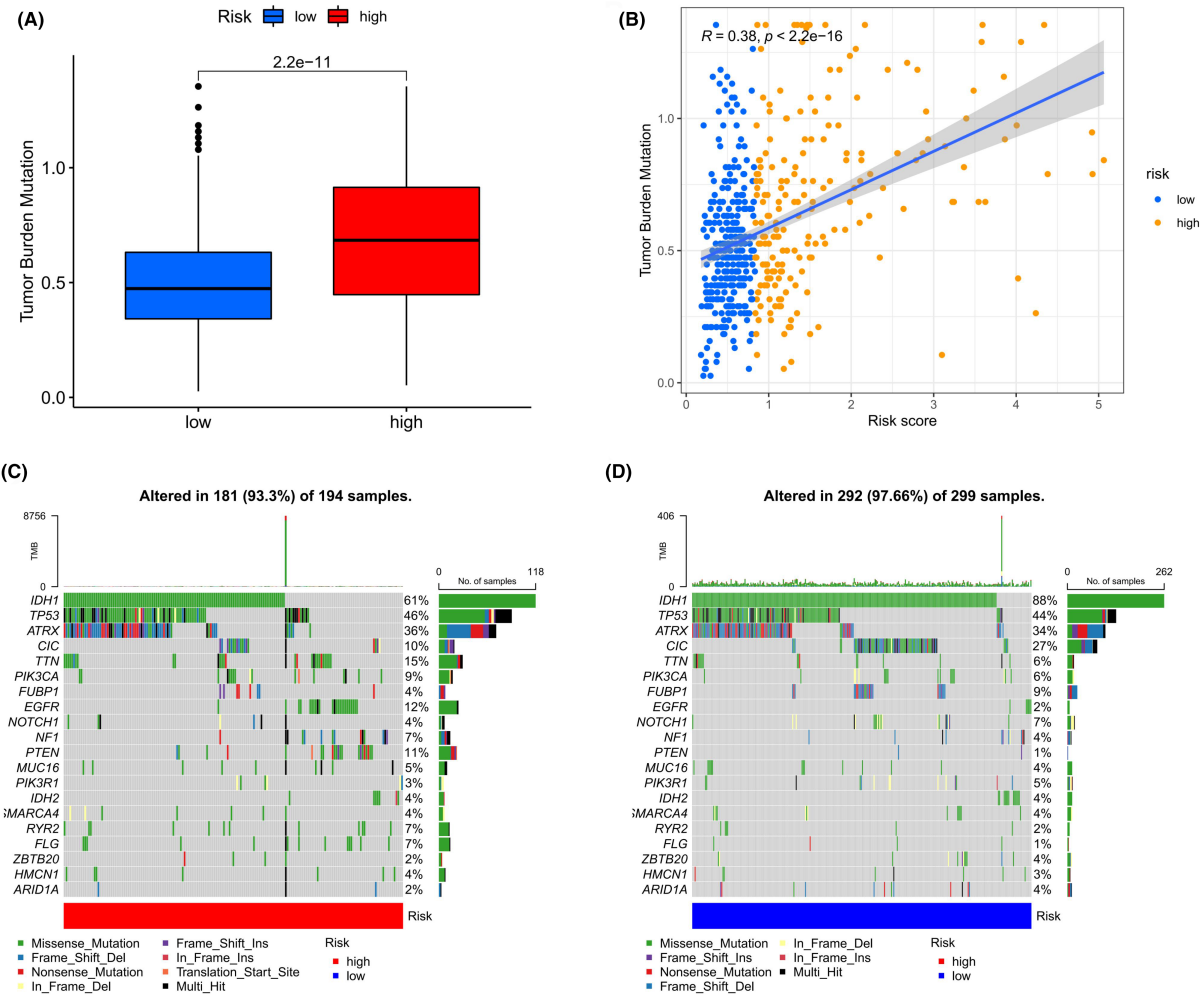
Analysis of mutational patterns linked to Risk Score stratification. (A) Comparison of tumour mutational burden (TMB) between patient groups stratified by elevated risk scores. (B) Association between the calculated risk score and the extent of TMB. (C, D) Comprehensive mutation profiles depicted via waterfall plots for patient groups categorized as high and low risk.

In our investigation within the TCGA‐LGG cohort, we focused on the interrelation between the established risk score and the tumour mutational burden (TMB). Our findings indicated an elevated TMB in the high‐risk category (Figure 8A,B). Delving deeper, we utilized waterfall plots to depict the mutation landscape in both risk strata. It was evident that mutations in genes such as IDH1, TP53 and ATRX were prevalent in both groups, with a notable reduction in IDH mutations in the high‐risk group (Figure 8C,D).

**FIGURE 9:**
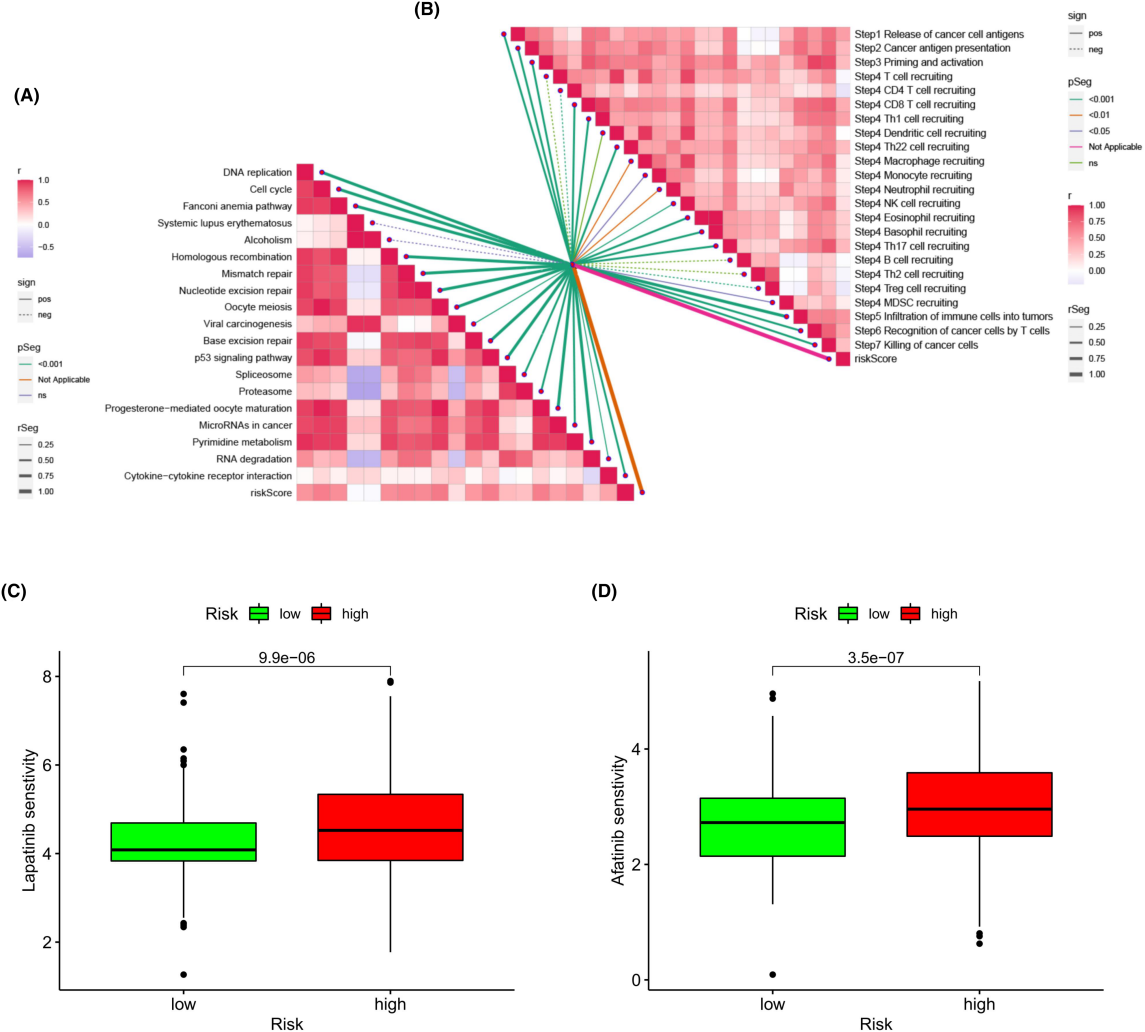
Assessment of Risk Score in relation to Immune Checkpoint Blockade (ICB) Efficacy. (A) Analysis of the association between risk score and immunotherapy response markers. (B) Examination of the correlation between the risk score and each phase of the tumour's immune response cycle. Additionally, sensitivity to chemotherapeutic drugs, lapatinib and afatinib, was evaluated for high‐risk and low‐risk groups, as indicated by IC50 values in (C) and (D), respectively.

Acknowledging the pivotal role of the immune microenvironment in shaping the efficacy of ICB therapies, we extended our analysis to examine the correlation between the risk score and various ICB response markers. This analysis revealed a unique pattern where only alcoholism exhibited a significant negative correlation with the risk score, whereas all other ICB response markers showed positive correlations (Figure 9A). Further, we embarked on a comprehensive correlation study to link the risk score with different phases of the tumour immune cycle. The risk score demonstrated a significant positive association with several critical stages, encompassing antigen release, presentation, priming and activation of immune responses, transportation of immune cells to tumour sites, and their infiltration into the tumour microenvironment (Figure 9B).

In our final phase of analysis, we utilized the ‘pRRophetic’ R package to assess the differential responsiveness to clinical therapeutic agents in high‐ and low‐risk groups. Focusing on glioma treatment, we identified two chemotherapeutic drugs, lapatinib and afatinib. Our analysis revealed that patients with higher risk scores exhibited higher IC50 values for these drugs, suggesting an enhanced sensitivity or responsiveness in the high‐risk group (Figure 9C,D).

### 3.8 ARHGAP family genes in single‐cell RNA sequencing

**FIGURE 10:**
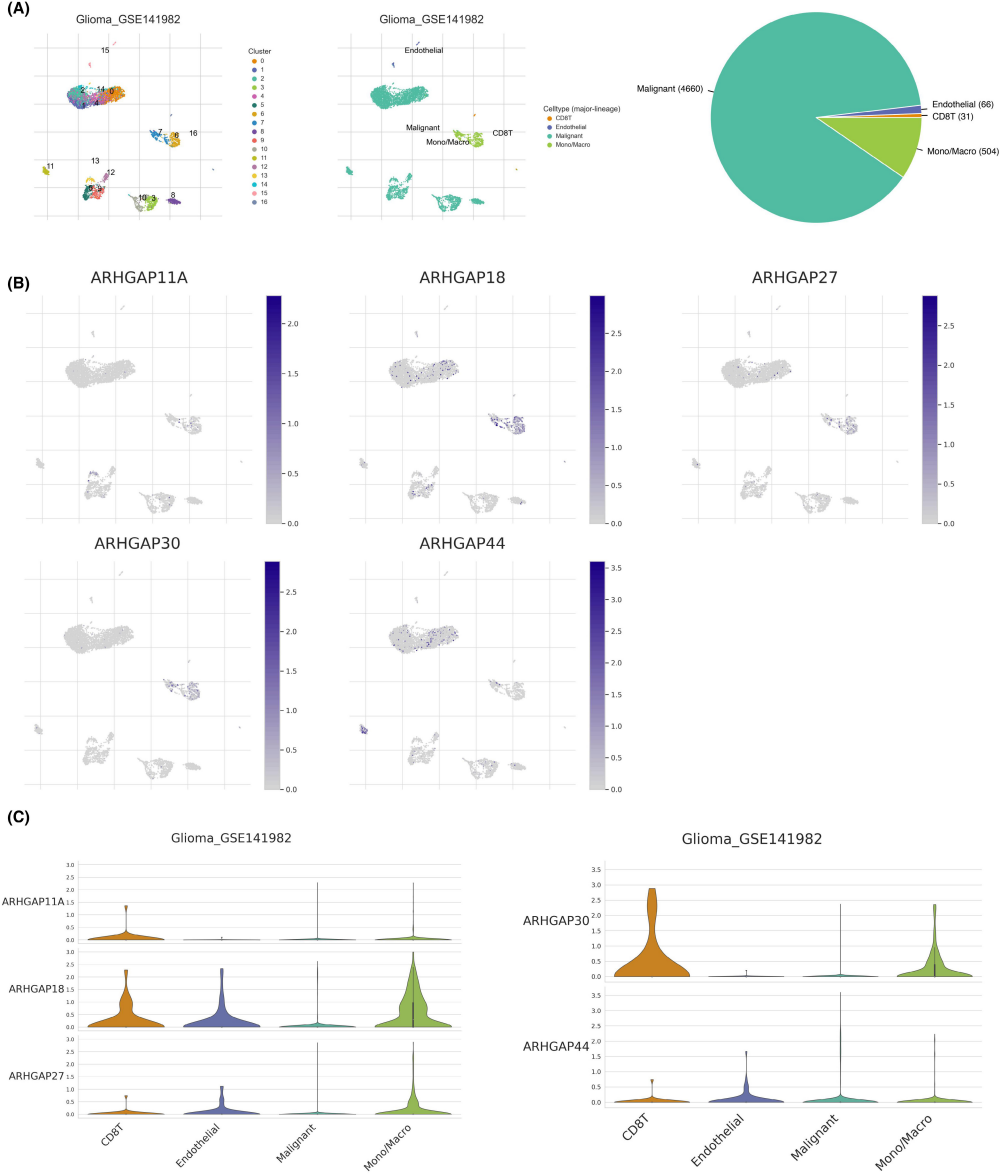
Analysis of ARHGAP Family gene expression in single‐cell RNA sequencing data. (A) Cell type categorization in dataset GSE141982, depicting the proportion of each cell type. (B, C) Exploration of the expression levels and distribution percentages of ARHGAP11A, ARHGAP18, ARHGAP27, ARHGAP30, and ARHGAP44 in various cell types.

In an in‐depth exploration of the tumour microenvironment (TME) in GBM, we analysed the expression patterns of selected ARHGAP family genes using the single‐cell dataset GSE141982 from the TISCH database. This dataset encompasses a diverse range of 16 cell populations and 4 subpopulations, providing a detailed view of the cellular composition in GBM (Figure 10A). Our analysis revealed that ARHGAP30 predominantly expressed in CD8^+^ T cells, while showing relatively low expression in GBM malignant cells. Conversely, ARHGAP18 was more prominently expressed in the monocyte‐derived macrophages, and ARHGAP44 was observed to be significantly expressed in endothelial cells. Notably, ARHGAP36 was absent in the samples under study (Figure 10B,C).

### 3.9 Cellular experiments

In the 6‐gene model developed for glioma prognosis, ARHGAP30 stood out due to its lower *p*‐value in univariate Cox analysis and its pronounced correlation with CD8^+^ T cells. It's noted in research that GBM often fails to initiate robust T‐cell inflammatory responses or experiences T‐cell exhaustion, making it less responsive to ICB therapies compared to tumours in peripheral organs like melanoma or breast cancer. The prevalence of terminally differentiated CD8^+^ tumour‐infiltrating lymphocytes (TILs) in GBM could be a contributing factor to this differing response to treatment.^24^ Consequently, ARHGAP30 was selected for further cellular function experiments to validate the prognostic model's accuracy.

**FIGURE 11:**
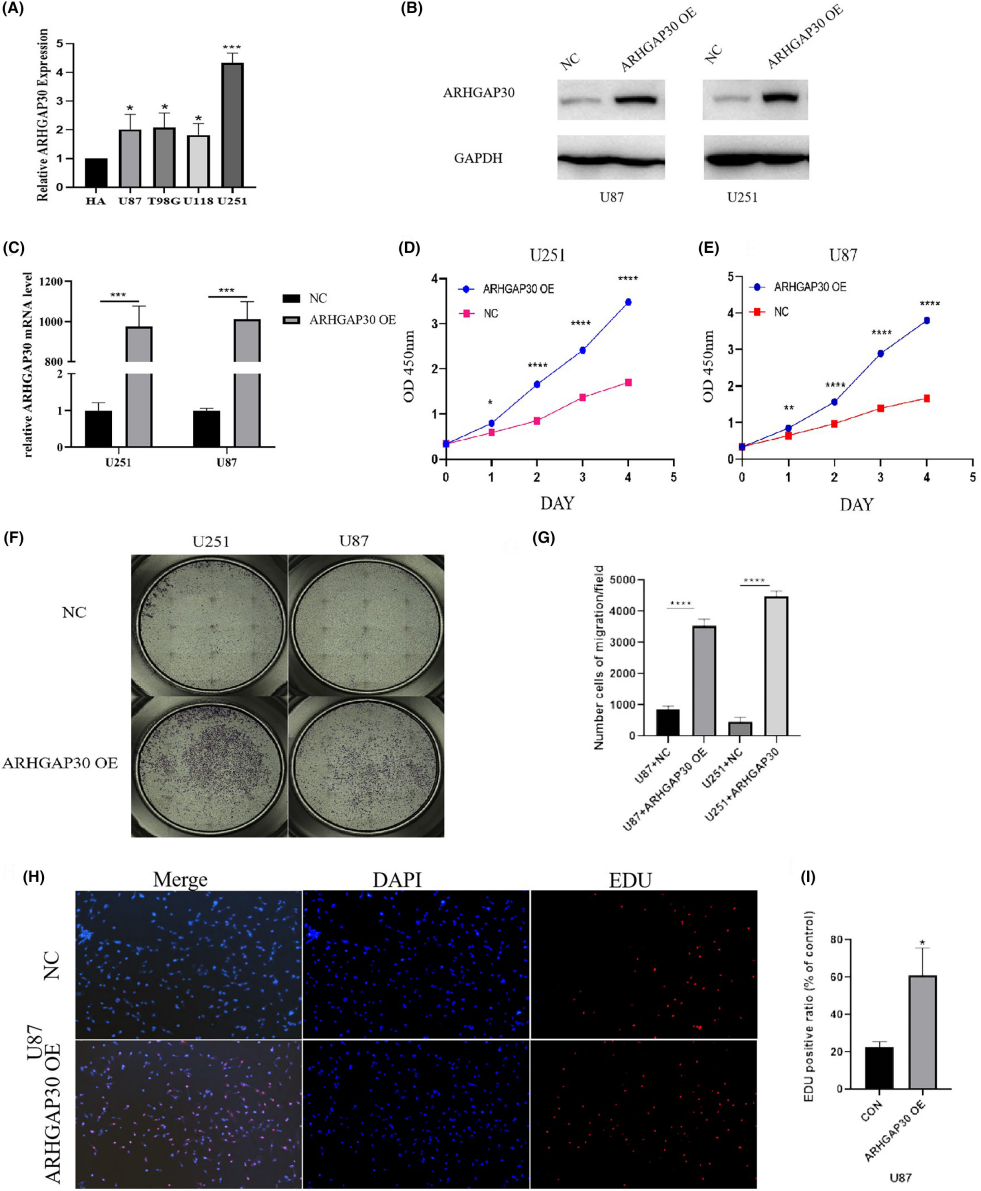
Investigating the impact of ARHGAP30 on glioma cell proliferation. (A) Assessment of ARHGAP30 mRNA levels in diverse glioma cell lines using RT‐qPCR. (B, C) Validation of ARHGAP30 overexpression in U87 and U251 cells through Western blot and qPCR analyses. (D, E) CCK‐8 assays were conducted to determine the proliferative capacity in U87 and U251 cells post ARHGAP30 overexpression compared to control (*n* = 3, *p* < 0.05). (F, G) Migration assays in U87 and U251 cells post‐transfection with ARHGAP30 or control vectors (*n* = 3, *p* < 0.05). (H, I) EdU incorporation assays performed in U87 cells to assess cell proliferation following ARHGAP30 overexpression versus control group (*n* = 3, *p* < 0.05). Significance denoted as *p < 0.05, **p < 0.01, ***p < 0.001, ****p < 0.0001.

To substantiate the link between ARHGAP30 upregulation and adverse outcomes in glioma, we conducted a series of cellular assays. RT‐qPCR analysis revealed varying ARHGAP30 expression levels across different cell lines including HA, U87, T98G, U118 and U251 (Figure 11A). Post‐transfection, a notable increase in ARHGAP30 expression was observed in U87 and U251 cells (Figure 11B,C). We then proceeded with cell proliferation assays on these transfected cells, employing CCK‐8 assay (Figure 11D,E), migration assay (Figure 11F,G), and EdU assay (Figure 11H,I). These experiments consistently demonstrated that ARHGAP30 significantly enhanced the proliferation of glioma cells, thereby corroborating the association between high ARHGAP30 expression and unfavourable glioma prognosis.

## 4 DISCUSSION

The management of glioma currently encompasses surgical intervention coupled with adjunctive therapies like radiation and chemotherapy. However, these strategies have not significantly improved the overall prognosis for glioma patients. Irrespective of the differentiation level, gliomas are notorious for their aggressive infiltration, rapid growth and high malignancy. These characteristics contribute to the dismal prognosis, elevated mortality rates and a substantial risk of tumour recurrence.^25^, ^26^

The progression of tumours to an invasive and metastatic state is marked by their ability to mobilize and invade surrounding tissues. This critical transition typically encompasses reductions in cellular adhesion, modifications of the extracellular matrix and the activation of mechanisms driven by the actin cytoskeleton.^27^ A notable phenomenon in this context is ‘anoikis’, a form of apoptosis that occurs when tumour cells lack appropriate extracellular matrix (ECM) interactions or adhere anomalously.^28^, ^29^ Essentially, cancers emerge from epithelial cells and convert them to cancerous through an elaborate process of genetic and physiological adjust, which is known as epithelial‐mesenchymal transition (EMT).^30^ This is often a change in the actin cytoskeleton's position.

Rho‐GTPases are like the backbone of protein family that includes GTP binding capability, important for numerous cell activities like cytoskeleton rearrangement, cell matrix adhesion and gene transactivating hormones rearrangement. Actin cytoskeleton is supported by these proteins by providing structural support to cell membranes and facilitating critical cellular processes.^31^ So, in fibroblast studies, it has been observed that the activation of Rho leads to the formation of stress fibres.^32^ Rho‐GTPase‐mediated cytoskeletal alterations are important for cell motility, which is integral to the study of fibroblast. According to some research papers, Rho‐GTPase control is very important when it comes to cancer building up. A decrease in the ARHGAP protein level may cause a rise in Rho‐GTPase activity which often leads to cancer. High expression levels of ARHGAP9, 15 and 30, for instance, have been correlated with better RFS and OS in breast cancer.^33^ Liu et al. discovered that ARGFAP15 hinders lung cancer cell proliferation and metastasis by repressing MMP2, MMP9 and VEGF expression via the deactivation of the STAT3 pathway.^34^ Remarkably, studies have shown that FOXP3 can regulate the expression of ARHGAP15 in glioma, with significant correlations between ARHGAP15 expression and glioma severity.^35^ The ARHGAP family genes are pivotal in orchestrating cytoskeletal remodelling and assembly, potentially playing significant roles in immune cell migration and thereby influencing tumour immune infiltration.

In this research, 10 genes from the ARHGAP family were identified via univariate Cox analysis. These genes were then used to stratify glioma patients in the CGGA cohort into two distinct subtypes through unsupervised clustering analysis. This stratification aimed to evaluate the role of ARHGAP family genes in glioma. We observed that, with the exception of ARHGAP36 and ARHGAP44, the remaining genes were predominantly expressed in subtype A. Notably, subtype A exhibited higher levels of the immune cell infiltration, which might be linked to the adverse prognosis observed in this group. Enrichment analysis further highlighted disparities in immune‐related pathways between subtypes A and B, suggesting that ARHGAP family genes could indirectly influence tumour immune infiltration and modulate immune response efficacy.

In this investigation, we focused on the prognostic relevance of the ARHGAP gene family in clinical oncology. Using advanced analytical methods, including Lasso‐penalized and multivariate Cox analyses, 6 ARHGAP genes—ARHGAP11A, ARHGAP18, ARHGAP27, ARHGAP30, ARHGAP36 and ARHGAP44—were identified as key independent prognostic factors. These genes collectively contribute to a comprehensive risk assessment model for cancer prognosis. Extensive research has highlighted the role of ARHGAP genes in the pathogenesis and progression of various cancers. Notably, ARHGAP11A has emerged as a significant biomarker linked with immune cell infiltration in cancers such as gastric cancer,^36^ hepatocellular carcinoma^37^ and lung adenocarcinoma.^38^ In breast cancer research, the interaction between ARHGAP18 and miR‐200b is particularly notable. miR‐200b's modulation of ARHGAP18 is observed to activate RhoA, leading to enhanced formation of adhesion sites and actin stress fibres, ultimately reducing tumour cell migration and metastatic potential.^39^

Yan et al. revealed the expression of ARHGAP36 in primary papillary thyroid cancer cells as well as in metastatic lesions and regulated their proliferation and migration through single‐cell sequencing.^40^ Through an extensive review, we found that ARHGAP27 and ARHGAP44 are hardly relevant in tumour research. The only studies found that ARHGAP44 as a downstream target of the mutant tumour suppressor protein p53 promotes tumour cell proliferation and migration.^41^ However, ARHGAP30 has been relatively well studied in different cancers, for example, ARHGAP30 inhibited the proliferation, migration and invasion of lung cancer cells^42^ and pancreatic cancer^43^ by inhibiting the Wnt/β‐catenin signalling pathway. In cervical cancer, ARHGAP30 may reduce ribosome biosynthesis and protein synthesis by promoting ubiquitination of NCL and thus tumour cell growth.^44^ However, there are no relevant basic studies on ARHGAP30 in glioma, and we found that by overexpressing ARHGAP30 in GBM cell lines, ARHGAP30 significantly promoted the proliferation of glioma cells. Just as ARHGAP30 exhibited biological changes in stromal remodelling, cell migration, invasion, and metastasis in other types of malignancies. Our findings may provide good evidence to explain ARHGAP30 in gliomas in EMT targeting and immunotherapy.

In this research, the prognostic impact of traditional factors like tumour grades, histological types, and IDH mutation status on glioma was evaluated, which are integral in determining treatment approaches including grading prognosis and selecting therapies like immunotherapy, radiotherapy and chemotherapy. However, the heterogeneity in clinicopathological characteristics among glioma patients indicates limitations in conventional staging methods. This study introduced a 6‐gene signature that effectively forecasts OS in glioma patients across both training and validation groups. High‐risk scores derived from this genetic model were statistically significant predictors of poorer prognosis. This gene signature independently predicted patient outcomes in the TCGA and CGGA cohorts, considering key clinical parameters such as tumour grades, ages and IDH mutation status. The model serves as a reliable surrogate for prognostication. Patients categorized under high‐risk scores generally presented with more aggressive tumour characteristics, including higher grades and IDH‐wild type status, indicative of the model's ability to identify poor prognostic features.

This is a detailed study about the effects of a particular genetic pattern in gliomas. We considered two types of patients, one with high risk and other with low risk. We looked at different aspects such as the immune system, the micro environment and the genetic changes to determine the impact of this signature on the prognosis of the patients. Research shows that there is an increase in CD8^+^ T‐cell infiltration in high‐risk category which match with our findings.^45^ According to our model, the gene ARHGAP30 appears to have a strong relation with the CD8^+^ T cells. Moreover, the high‐risk group showed a higher tumour mutational burden which indicates that there is more scope for the body's immune system to recognize and attack the cancerous cells. This pattern suggests that ICI therapy can be more effective in these patients.^46^ These new discoveries in glioma treatment are really exciting as they are changing the way we think about treating this type of cancer. The emphasis on immunotherapeutic methods means that we are moving towards a more personalized approach to treatment, which is great news for patients. There are some new techniques like photoimmunotherapy that are showing potential for altering the way the immune system fights against brain tumours.^47^ But the difficulty of the intrinsically immunosuppressive environment of GBM restricts the effectiveness of standard immunotherapy practices.^48^ In the high‐risk group, immature macrophages are expressed at a higher level, but there is not more expression of activated macrophages to play a role in the immune response. We believe that this may be due to the more common phenomenon of immune silence in malignant gliomas with higher degrees of malignancy. ARHGAP18 is more significantly expressed in monocyte‐derived macrophages. This suggests that ARHGAP18 may be related to the immune response of monocyte‐derived macrophages in tumours. Our research determined the relationship between the risk score of ARHGAP family genes and the immune response to ICB in gliomas. Our study describes how to construct a predictive nomogram using precise biomarkers and gene signatures to estimate the prognosis of patients with glioma. This work not only optimizes the ability of clinicians to develop personalized treatment strategies but also provides more treatment options and the potential to improve survival outcomes for patients with glioma.^49^ Our research has been validated through DCA, indicating that this tool may assist in guiding clinical decisions, especially in predicting patient survival rates and developing treatment plans.

In summary, the proposed 6‐gene prognostic model demonstrates promising potential in forecasting the survival outcomes for patients with glioma. This model offers valuable insights for clinicians to tailor treatment approaches more effectively. The DCA suggests that the nomogram, formulated on the basis of this gene signature, could be particularly beneficial for glioma patients with a more challenging prognosis, providing guidance within a shorter timeframe. Columnar visualizations derived from this model have the potential to aid physicians in designing individualized, immune‐focused therapeutic strategies in clinical settings. Nonetheless, the study has certain limitations. Primarily, the findings are predominantly based on bioinformatic analyses with only ARHGAP30 undergoing detailed cellular experimentation. Further investigative efforts are required to elucidate the roles of other ARHGAP family genes in glioma. Moreover, the validity of the ARHGAP family gene‐based biomarker needs to be established through extensive clinical trials to reinforce our conclusions.

However, this study has some limitations. We did not include more experimental studies to further validate the prognostic value of the risk model or to investigate its regulatory mechanisms.

## 5 CONCLUSION

In conclusion, we identified a novel genetic marker that influences the prognosis of glioma within the ARHGAP family. This finding holds substantial importance in the realm of precise glioma treatment and genetic diagnosis.

## AUTHOR CONTRIBUTIONS

**Jin Huang:** Conceptualization (equal); data curation (equal); formal analysis (equal); funding acquisition (supporting); writing – original draft (equal). **Gaosong Wang:** Conceptualization (equal); formal analysis (equal). **Jiahao Zhang:** Data curation (equal); methodology (equal); writing – original draft (equal). **Yuankun Liu:** Formal analysis (equal); methodology (equal). **Yifan Shen:** Formal analysis (equal). **Gengjing Chen:** Supervision (equal); validation (equal); writing – review and editing (equal). **Wei Ji:** Formal analysis (equal); methodology (equal). **Junfei Shao:** Funding acquisition (equal); validation (equal); writing – original draft (equal).

## FUNDING INFORMATION

This work was supported by the Wuxi Taihu Lake Talent Plan, Supports for Leading Talents in Medical and Health Profession (DJYX‐2020), the National Natural Science Foundation of China (82172955), the scientific research project of Wuxi health commission (2020ZHYB16), the Youth project of Wuxi commission of Health (Q202133) and the Basic Research Project of Wuxi Science and Technology Bureau (K20221024).

## CONFLICT OF INTEREST STATEMENT

The authors claim that we performed this research without any business or financial relationships that could be interpreted as potential conflicts of interest.

## Data Availability

Publicly available datasets were analysed in this study. This data can be found here: The datasets analysed in the current study are available in the TCGA repository (http://cancergenome.nih.gov/), CGGA (http://www.cgga.org.cn/) and Genotype‐Tissue Expression (GTEx) website (https://www.gtexportal.org/). All raw data and original images can be found in the jianguoyun (https://www.jianguoyun.com/p/DRGxdpcQ0pH7ChjlltoEIAA).

## References

[1] Schaff LR , Mellinghoff IK . Glioblastoma and other primary brain malignancies in adults: a review. JAMA. 2023;329(7):574‐587. doi:10.1001/jama.2023.0023 36809318 PMC11445779

[2] Horbinski C , Berger T , Packer RJ , Wen PY . Clinical implications of the 2021 edition of the WHO classification of central nervous system tumours. Nat Rev Neurol. 2022 Sep;18(9):515‐529. doi:10.1038/s41582-022-00679-w 35729337

[3] Luo C , Song K , Wu S , et al. The prognosis of glioblastoma: a large, multifactorial study. Br J Neurosurg. 2021;35(5):555‐561. doi:10.1080/02688697.2021.1907306 34236262

[4] Louis DN , Perry A , Wesseling P , et al. The 2021 WHO classification of tumors of the central nervous system: a summary. Neuro‐Oncology. 2021;23(8):1231‐1251. doi:10.1093/neuonc/noab106 34185076 PMC8328013

[5] Anand U , Dey A , Chandel AKS , et al. Cancer chemotherapy and beyond: current status, drug candidates, associated risks and progress in targeted therapeutics. Genes Dis. 2022;10(4):1367‐1401. doi:10.1016/j.gendis.2022.02.007 37397557 PMC10310991

[6] Han S , Liu Y , Cai SJ , et al. IDH mutation in glioma: molecular mechanisms and potential therapeutic targets. Br J Cancer. 2020;122(11):1580‐1589. doi:10.1038/s41416-020-0814-x 32291392 PMC7250901

[7] van der Voort SR , Incekara F , Wijnenga MMJ , et al. Combined molecular subtyping, grading, and segmentation of glioma using multi‐task deep learning. Neuro‐Oncology. 2023;25(2):279‐289. doi:10.1093/neuonc/noac166 35788352 PMC9925710

[8] Gao Y , Bado I , Wang H , Zhang W , Rosen JM , Zhang XH . Metastasis organotropism: redefining the congenial soil. Dev Cell. 2019;49(3):375‐391. doi:10.1016/j.devcel.2019.04.012. 31063756 PMC6506189

[9] Steeg PS . Targeting metastasis. Nat Rev Cancer. 2016;16(4):201‐218. doi:10.1038/nrc.2016.25 27009393 PMC7055530

[10] Huse M . Mechanical forces in the immune system. Nat Rev Immunol. 2017;17(11):679‐690. doi:10.1038/nri.2017.74 28757604 PMC6312705

[11] Durrant TN , van den Bosch MT , Hers I . Integrin αIIbβ3 outside‐in signaling. Blood. 2017;130(14):1607‐1619. doi:10.1182/blood-2017-03-773614 28794070 PMC5672329

[12] Moore AR , Rosenberg SC , McCormick F , Malek S . RAS‐targeted therapies: is the undruggable drugged? Nat Rev Drug Discov. 2020;19(8):533‐552. doi:10.1038/s41573-020-0068-6 32528145 PMC7809886

[13] Cartier A , Hla T . Sphingosine 1‐phosphate: lipid signaling in pathology and therapy. Science. 2019;366(6463):eaar5551. doi:10.1126/science.aar5551 31624181 PMC7661103

[14] Post A , Pannekoek WJ , Ross SH , Verlaan I , Brouwer PM , Bos JL . Rasip1 mediates Rap1 regulation of rho in endothelial barrier function through ArhGAP29. Proc Natl Acad Sci USA. 2013;110(28):11427‐11432. doi:10.1073/pnas.1306595110 23798437 PMC3710801

[15] Yang L , Xu Q , Li J . Prognostic impact of ARHGAP_43_(SH3BP1) in acute myeloid leukemia. J Formos Med Assoc. 2024. S0929‐6646(24)00186‐4. Epub ahead of print. doi:10.1016/j.jfma.2024.04.002 38582737

[16] Komatsu M , Ichikawa H , Chiwaki F , et al. ARHGAP‐RhoA signaling provokes homotypic adhesion‐triggered cell death of metastasized diffuse‐type gastric cancer. Oncogene. 2022;41(43):4779‐4794. doi:10.1038/s41388-022-02469-6 36127398

[17] Leek JT , Johnson WE , Parker HS , Jaffe AE , Storey JD . The sva package for removing batch effects and other unwanted variation in high‐throughput experiments. Bioinformatics. 2012;28(6):882‐883. doi:10.1093/bioinformatics/bts034 22257669 PMC3307112

[18] Hänzelmann S , Castelo R , Guinney J . GSVA: gene set variation analysis for microarray and RNA‐seq data. BMC Bioinformatics. 2013;14:7. doi:10.1186/1471-2105-14-7 23323831 PMC3618321

[19] Wilkerson MD , Hayes DN . ConsensusClusterPlus: a class discovery tool with confidence assessments and item tracking. Bioinformatics. 2010;26(12):1572‐1573. doi:10.1093/bioinformatics/btq170 20427518 PMC2881355

[20] Newman AM , Liu CL , Green MR , et al. Robust enumeration of cell subsets from tissue expression profiles. Nat Methods. 2015;12(5):453‐457. doi:10.1038/nmeth.3337 25822800 PMC4739640

[21] Vickers AJ , Cronin AM , Elkin EB , Gonen M . Extensions to decision curve analysis, a novel method for evaluating diagnostic tests, prediction models and molecular markers. BMC Med Inform Decis Mak. 2008;8:53. doi:10.1186/1472-6947-8-53 19036144 PMC2611975

[22] Sun D , Wang J , Han Y , et al. TISCH: a comprehensive web resource enabling interactive single‐cell transcriptome visualization of tumor microenvironment. Nucleic Acids Res. 2021;49(D1):D1420‐D1430. doi:10.1093/nar/gkaa1020 33179754 PMC7778907

[23] Reiter JG , Baretti M , Gerold JM , et al. An analysis of genetic heterogeneity in untreated cancers. Nat Rev Cancer. 2019;19(11):639‐650. doi:10.1038/s41568-019-0185-x 31455892 PMC6816333

[24] Wang H , Xu T , Huang Q , Jin W , Chen J . Immunotherapy for malignant glioma: current status and future directions. Trends Pharmacol Sci. 2020;41(2):123‐138. doi:10.1016/j.tips.2019.12.003 31973881

[25] Lai G , Li K , Deng J , Liu H , Xie B , Zhong X . Identification and validation of a gene signature for lower‐grade gliomas based on pyroptosis‐related genes to predict survival and response to immune checkpoint inhibitors. J Healthc Eng. 2022;2022:8704127. doi:10.1155/2022/8704127 35535221 PMC9078805

[26] Shen Y , Chi H , Xu K , et al. A novel classification model for lower‐grade glioma patients based on pyroptosis‐related genes. Brain Sci. 2022;12(6):700. doi:10.3390/brainsci12060700 35741587 PMC9221219

[27] Rankin EB , Giaccia AJ . Hypoxic control of metastasis. Science. 2016;352(6282):175‐180. doi:10.1126/science.aaf4405 27124451 PMC4898055

[28] Boudreau NJ , Jones PL . Extracellular matrix and integrin signalling: the shape of things to come. Biochem J. 1999;339:481‐488. 10215583 PMC1220180

[29] Chiarugi P , Giannoni E . Anoikis: a necessary death program for anchorage‐dependent cells. Biochem Pharmacol. 2008;76(11):1352‐1364. doi:10.1016/j.bcp.2008.07.023 18708031

[30] Williams ED , Gao D , Redfern A , Thompson EW . Controversies around epithelial‐mesenchymal plasticity in cancer metastasis. Nat Rev Cancer. 2019;19(12):716‐732. doi:10.1038/s41568-019-0213-x 31666716 PMC7055151

[31] Zheng Y , Pan D . The hippo signaling pathway in development and disease. Dev Cell. 2019;50(3):264‐282. doi:10.1016/j.devcel.2019.06.003 31386861 PMC6748048

[32] Nobes CD , Hall A . Rho, rac, and cdc42 GTPases regulate the assembly of multimolecular focal complexes associated with Actin stress fibers, lamellipodia, and filopodia. Cell. 1995;81(1):53‐62. doi:10.1016/0092-8674(95)90370-4 7536630

[33] Chen WX , Lou M , Cheng L , et al. Bioinformatics analysis of potential therapeutic targets among ARHGAP genes in breast cancer. Oncol Lett. 2019;18(6):6017‐6025. doi:10.3892/ol.2019.10949 31788076 PMC6864933

[34] Liu ZD , Mou ZX , Che XH , et al. ARHGAP15 regulates lung cancer cell proliferation and metastasis via the STAT3 pathway. Eur Rev Med Pharmacol Sci. 2019;23(13):5840‐5850. doi:10.26355/eurrev_201907_18326 31298335

[35] Sun Z , Zhang B , Wang C , et al. Forkhead box P3 regulates ARHGAP15 expression and affects migration of glioma cells through the Rac1 signaling pathway. Cancer Sci. 2017;108(1):61‐72. doi:10.1111/cas.13118 27862679 PMC5276829

[36] Fan B , Ji K , Bu Z , et al. ARHGAP11A is a prognostic biomarker and correlated with immune infiltrates in gastric cancer. Front Mol Biosci. 2021;8:720645. doi:10.3389/fmolb.2021.720645 34733886 PMC8558302

[37] Dai B , Zhang X , Shang R , et al. Blockade of ARHGAP11A reverses malignant progress via inactivating Rac1B in hepatocellular carcinoma. Cell Commun Signal. 2018;16:99. doi:10.1186/s12964-018-0312-4 30545369 PMC6293628

[38] Li L , Peng M , Xue W , et al. Integrated analysis of dysregulated long non‐coding RNAs/microRNAs/mRNAs in metastasis of lung adenocarcinoma. J Transl Med. 2018;16(1):372. doi:10.1186/s12967-018-1732-z 30587197 PMC6307237

[39] Humphries B , Wang Z , Li Y , Jhan JR , Jiang Y , Yang C . ARHGAP18 downregulation by miR‐200b suppresses metastasis of triple‐negative breast cancer by enhancing activation of RhoA. Cancer Res. 2017;77(15):4051‐4064. doi:10.1158/0008-5472.CAN-16-3141 28619708

[40] Yan T , Qiu W , Song J , Fan Y , Yang Z . ARHGAP36 regulates proliferation and migration in papillary thyroid carcinoma cells. J Mol Endocrinol. 2021;66(1):1‐10. doi:10.1530/JME-20-0230 33112823

[41] Xu J , Jiao J , Xu W , et al. Mutant p53 promotes cell spreading and migration via ARHGAP44. Sci China Life Sci. 2017;60(9):1019‐1029. doi:10.1007/s11427-016-9040-8 28527113

[42] Mao X , Tong J . ARHGAP30 suppressed lung cancer cell proliferation, migration, and invasion through inhibition of the Wnt/β‐catenin signaling pathway. Onco Targets Ther. 2018;11:7447‐7457. doi:10.2147/OTT.S175255 30425532 PMC6204876

[43] Zhou Y , Hua Z , Zhu Y , et al. Upregulation of ARHGAP30 attenuates pancreatic cancer progression by inactivating the β‐catenin pathway. Cancer Cell Int. 2020;20:225. doi:10.1186/s12935-020-01288-7 32536813 PMC7288688

[44] Wu A , Lin L , Li X , et al. Overexpression of ARHGAP30 suppresses growth of cervical cancer cells by downregulating ribosome biogenesis. Cancer Sci. 2021;112(11):4515‐4525. doi:10.1111/cas.15130 34490691 PMC8586670

[45] Luo C , Liu Z , Ye W , Liu F . Immune infiltration‐related signature predicts risk stratification and immunotherapy efficacy in grade II and III gliomas. Front Cell Dev Biol. 2021;9:756005. doi:10.3389/fcell.2021.756005 34805164 PMC8603377

[46] Chan TA , Yarchoan M , Jaffee E , et al. Development of tumor mutation burden as an immunotherapy biomarker: utility for the oncology clinic. Ann Oncol. 2019;30(1):44‐56. doi:10.1093/annonc/mdy495 30395155 PMC6336005

[47] Mączyńska J , Raes F , Da Pieve C , et al. Triggering anti‐GBM immune response with EGFR‐mediated photoimmunotherapy. BMC Med. 2022;20(1):16. doi:10.1186/s12916-021-02213-z 35057796 PMC8780306

[48] Weiss T , Puca E , Silginer M , et al. Immunocytokines are a promising immunotherapeutic approach against glioblastoma. Sci Transl Med. 2020;12(564):eabb2311. doi:10.1126/scitranslmed.abb2311 33028706

[49] Wang Z , Sun W , Hua R , Wang Y , Li Y , Zhang H . Promising dawn in tumor microenvironment therapy: engineering oral bacteria. Int J Oral Sci. 2024;16(1):24. doi:10.1038/s41368-024-00282-3 38472176 PMC10933493

